# First Edition Special Issue on “Cellular and Molecular Mechanisms in Immune Regulation”

**DOI:** 10.3390/cells14201614

**Published:** 2025-10-17

**Authors:** Fabio R. Santori, Natalia B. Ivanova

**Affiliations:** Center for Molecular Medicine, University of Georgia, 325 Riverbend Road, Athens, GA 30602, USA

In 1974, Niels K. Jerne proposed one of the first theories of immunoregulation [1]. Indeed, in his prescient paper he projected that the decades of 1970–1990 would be centered on systemic immune regulation mediated by cells cooperating with each other in multicellular networks [1]. It has been suggested that this self-regulating network of immune cells would function as an “immune sensory brain” that interprets changes in the body epitope repertoire recognized by the antigen-specific receptors of B and T cells [2]. Treating the immune system as an “immune sensory brain” suggests that it is flexible and that it could be “trained” to respond to specific stimuli and learn from its experiences. This could lead to changes in “immune sensory perception” and interpretation of bodily states. In practical terms it would mean that we could train the immune system to recognize tumors as targets for removal. This would include tumors that are naturally not immunogenic, such as those characterized as immune deserts [3].

Jerne’s predictions are now coming to fruition. The importance of immunoregulation to medicine was recognized by the 2025 Nobel Prize in Medicine and Physiology awarded to Mary E. Brunknow, Fred Ramsdell and Shimon Sakaguchi. Although we are still analyzing the regulation of the immune system in parts, with T cells, B cells and macrophages being mostly studied separately, one could foresee that the next few decades will see an explosion of systemic multi-cellular network approaches being used in the study and treatment of cancer, autoimmune diseases, allergies and infections. The first edition of the Special Issue on “Cellular and Molecular Mechanisms in Immune Regulation” reflects the current state of the field of immune regulation and its movement towards a systemic multi-cellular approach.

As an opening note, we start with a systematic review of the latest advances in the molecular mechanisms of immune regulation by Dr. Arneth [4], which is followed by the work of Lodde and colleagues which analyzes the use of circular RNAs as potential biomarkers of immune function in multiple sclerosis [5]. The article by Sim and colleagues explores the effect of X chromosome loss (Turner syndrome) on immunoregulation [6]. Despite the small sample of their patient cohort, their results are intriguing, suggesting that at least 43 immunoregulatory genes may be affected by the loss of chromosome X. We are looking forward to larger studies that will strengthen and expand these results.

The next focus of the Special Issue is on autoimmunity, and the article by Ferreira-Hermosillo and colleagues provides us a glimpse of circulating T cell subsets in Type 1 diabetes [7]. The role of lipid biosynthesis and catabolism in immune regulation is highlighted in the review by Jiani Xing, Takese Mckenzie and Jian Hu, where the authors focus on the role of lipid-laden microglia in inflammation that accompanies neurodegenerative diseases as well as the healing processes following spinal cord injuries and strokes [8]. The article by Memida and colleagues explores the role of IL-10-producing regulatory B cells in the generation of M2 anti-inflammatory macrophages [9]. The review by Sorab Ahmadivand, Robert Fux and Dušan Palič highlights recent advances in the field of immune cell communication focusing on the interaction of T follicular helper cells and B cells in viral infections and vaccine design [10]. The role of immune regulation in cancer is explored by two research articles. Eljilany and colleagues et al. [11] report the analyses of immune populations in patients with ovarian, bladder, pancreatic cancer and melanoma. Indeed, these authors report a beneficial effect of the increase of tissue resident memory T cells (TRMs) in the therapeutic outcome of patients treated with immune checkpoint inhibitors. This leads us to the review by Montgomery and colleagues [12] on the role of TRMs in the control of metastasis. TRMs are, indeed, a promising avenue for cancer therapy in metastatic disease. However, our models are currently limited to lung metastasis and more studies are needed to evaluate and promote the role of TRMs in the treatment of metastasis in brain, bone marrow and liver [12].

The Special Issue concludes with two articles highlighting the interactions between the environment and immune system, specifically on the role of oxygen tension and ultraviolet light (UV). The key is to understand how the homeostasis between organism and immune system is maintained with a changing environment. Here, the role of oxygen plays a key role. The work by Peter and colleagues [13] focuses on the role of oxygen and suggests that under physiological tissue concentrations of oxygen (4% O_2_, normoxia), dendritic cells shift towards a tolerogenic phenotype which contrasts with a pro-inflammatory phenotype observed under hyperoxia (21% O_2_) or hypoxia (<2% O_2_) [14]. Similarly, the skin in our body is in constant contact with light. UV light can cause mutations and dimerization of DNA but it is also required for production of vitamin D. The review by Gelare Ghajar-Rahimi, Nabiha Yusuf and Hui Xu [15] highlights the recent advances in our understanding of the role and mechanism by which UV can promote a tolerogenic phenotype in dendritic cell populations in the skin. The authors suggest that the tolerogenic effect of UV has both beneficial and pathologic aspects; thus, a delicate balance is necessary to maintain bodily homeostasis.

The first edition of the Special Issue on “Cellular and Molecular Mechanisms in Immune Regulation” gives a glimpse of the multi-cellular immunoregulatory networks implicated in autoimmune diseases, infection and cancer, as well as during normal interactions with the environment. Many topics were left unexplored, such as the regulation of pain and sensory nerve endings during immune responses, the interplay between the nervous system and the development of the immune system during ontogeny. We expect to cover many of these topics in the upcoming second edition of “Cellular and Molecular Mechanisms in Immune Regulation”.
